# Combining patient reported outcomes and EHR data to understand population level treatment needs: correcting for selection bias in the migraine signature study

**DOI:** 10.1186/s41687-021-00401-2

**Published:** 2021-12-18

**Authors:** Walter F. Stewart, Xiaowei Yan, Alice Pressman, Alice Jacobson, Shruti Vaidya, Victoria Chia, Dawn C. Buse, Richard B. Lipton

**Affiliations:** 1 Medcurio Inc., Oakland, CA 94618 USA; 2 Sutter Health Center for Health Systems Research, 2121 N. California Blvd., Ste. 310, Walnut Creek, CA 94596 USA; 3 Amgen Inc., Thousand Oaks, CA 91320 USA; 4 Montefiore Medical Center, Bronx, NY 10467 USA; 5 Albert Einstein College of Medicine, Bronx, NY 10461 USA

**Keywords:** Non-response bias, Electronic health records, Migraine disability, Prescription medications

## Abstract

**Background:**

Electronic health records (EHR) data can be used to understand population level quality of care especially when supplemented with patient reported data. However, survey non-response can result in biased population estimates. As a case study, we demonstrate that EHR and survey data can be combined to estimate primary care population prescription treatment status for migraine stratified by migraine disability, without and with adjustment for survey non-response bias. We selected disability as it is associated with survey participation and patterns of prescribing for migraine.

**Methods:**

A stratified random sample of Sutter Health adult primary care (PC) patients completed a digital survey about headache, migraine, and migraine related disability. The survey data from respondents with migraine were combined with their EHR data to estimate the proportion who had prescription orders for acute or preventive migraine treatments. Separate proportions were also estimated for those with mild disability (denoted “mild migraine”) versus moderate to severe disability (denoted mod-severe migraine) without and with correction, using the inverse propensity weighting method, for non-response bias. We hypothesized that correction for non-response bias would result in smaller differences in proportions who had a treatment order by migraine disability status.

**Results:**

The response rate among 28,268 patients was 8.2%. Among survey respondents, 37.2% had an acute treatment order and 16.8% had a preventive treatment order. The response bias corrected proportions were 26.2% and 11.6%, respectively, and these estimates did not differ from the total source population estimates (i.e., 26.4% for acute treatments, 12.0% for preventive treatments), validating the correction method. Acute treatment orders proportions were 32.3% for mild migraine versus 37.3% for mod-severe migraine and preventive treatment order proportions were 12.0% for mild migraine and 17.7% for mod-severe migraine. The response bias corrected proportions for acute treatments were 24.8% for mild migraine and 26.6% for mod-severe migraine and the proportions for preventive treatment were 8.1% for mild migraine and 12.0% for mod-severe migraine.

**Conclusions:**

In this study, we combined survey data with EHR data to better understand treatment needs among patients diagnosed with migraine. Migraine-related disability is directly related to preventive treatment orders but less so for acute treatments. Estimates of treatment status by self-reported disability status were substantially over-estimated among those with moderate to severe migraine-related disability without correction for non-response bias.

**Supplementary Information:**

The online version contains supplementary material available at 10.1186/s41687-021-00401-2.

## Background

Quality of care is often assessed using Electronic health records (EHR) data or survey data. For underdiagnosed conditions, EHR data do not capture the undiagnosed cases and do not provide a means to consistently assess symptom severity or functional impact. Survey data with a diagnostic screener can capture undiagnosed cases and offers a direct means of documenting patient symptoms and functional status. Survey data are often limited, however, by modest participation rates and the potential for response bias. Healthcare systems are increasingly combining EHR and survey data to better evaluate population level gaps in treatment, but without recognizing how response bias can influence results [1, 2].

Herein, we present migraine as a use-case to demonstrate that the combined use of EHR data and survey data facilitates a better understanding of population health needs and overcomes the response bias common to traditional population-based surveys. Migraine is a prevalent, often disabling chronic disease which exemplifies other symptomatic and burdensome diseases where people may not seek care and those that do seek care may not be diagnosed or receive an appropriate treatment [3–13]. Survey data indicate that variation in use of acute and preventive medications is directly linked to migraine-related disability and to associated comorbidities [5–7, 12, 14]. However, survey data for migraine are also prone to non-response and reporting biases in ways that directly influence estimates of migraine severity and prescription medications use whether a survey is done within a healthcare system or in the general population [15–20]. Moreover, response probability is associated with the severity of the disease being studied, and tends to be lower among those with lower education and socio-economic status (SES) and who are non-Caucasian race, younger age, and male gender [15–18]. Finally, the validity of self-report also varies by some of these same factors, as education and SES levels influence ability to interpret questions and response options [19–21].

Studies of migraine prescription drug use that rely on EHR data or medical claims do not have the concerns of non-response and recall bias, but have other limitations. Medication claims or EHR data are limited to patients who had sought care for migraine, underestimating the size of the population with migraine, and most EHR data document medication orders, not whether the patient actually obtained the medication. Evidence suggests that approximately 20% of first prescriptions are not adjudicated [22–25]. Neither medication claims nor EHR data capture information on those with undiagnosed migraine. EHR data generally does not systematically include information on migraine patient reported outcomes (PROs), including pain and symptom intensity, days with headache, or associated disability, precluding population health assessment of need for care. More generally, few studies combine EHR data with patient reported outcomes to gain a more comprehensive understanding of patient needs versus the care they receive [26].

The objective of this analysis of data from the Migraine Signature Study (MSS) was to demonstrate a use-case that combines the strengths of EHR and survey data to accurately understand population health level needs and, in particular, the relation of migraine related disability and prescription medication care. We leveraged the availability of EHR data on all patients to both quantify population level prescription care and to adjust for non-response bias from those invited to participate in the complementary MSS survey on migraine diagnostic questions and migraine disability status.

## Methods

Longitudinal EHR data were obtained on all adult primary care (PC) patients from the Sutter Health System. Survey data on headache and migraine experience were obtained on a stratified random sample of patients. We used EHR data to specifically estimate the proportion of survey respondents with migraine who were prescribed acute or preventive migraine treatments without and with correction for non-response bias. The Sutter Health Institutional Review Board approved the study.

### Sources of data

The study population was comprised of adult PC patients who sought care from Sutter Health, a large, not-for-profit integrated healthcare network serving 22 counties in northern California. The Sutter Health Medical Network includes 1200 primary care providers, 126 neurologists, and a diversity of other ambulatory and inpatient care services. Sutter Heath uses a single instance of EpicCare (Epic) EHR.

#### EHR data

EHR data are organized around encounters and activities that include ambulatory, inpatient, emergency department, telephone, and video, among others. For this study, an individual was defined as an adult PC patient if they had at least one office visit to a PC department during the 5-year study period from 1/1/2013 to 12/31/2017 and were 18–75 years of age sometime during this time period. EHR data were extracted on eligible PC patients for all encounters occurring during the study period and included encounter type, date, and diagnosis (i.e., ICD-9 and ICD-10 codes for primary and secondary diagnosis), and, separately, medication ordered and diagnostic indication for the order.

**Fig. 1:**
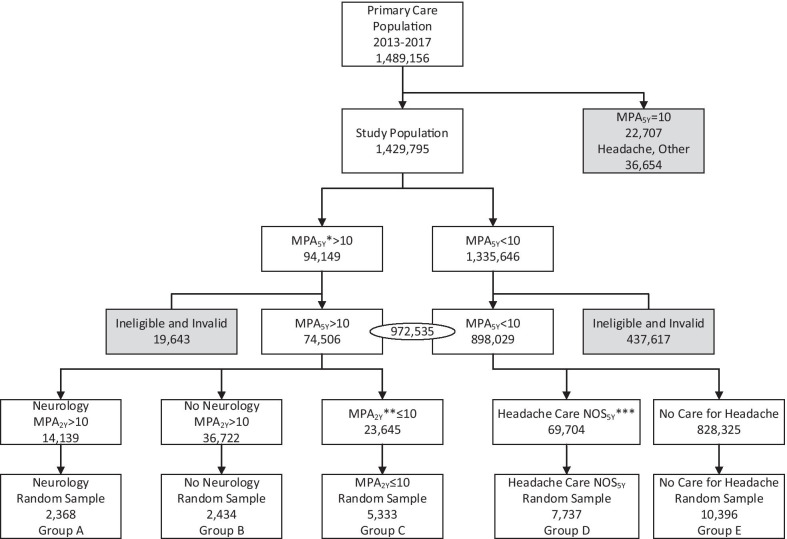
Flowchart of population selection and sampling based on EHR data. MPA_5Y_ > 10 Patient is likely to have had migraine at some point in the past 5 years. **MPA_2Y _> 10 Patient is likely to have used care for migraine in the past 2 years. ***Headache Care NOS_5Y_: Care for Headache Not Otherwise Specified during the study period

Migraine Probability Algorithm (MPA) scores were calculated from EHR data on all patients to estimate the probability of having clinically diagnosed migraine [27]. The MPA score was validated in an independent health system, and, for a cut-point of MPA > 10, sensitivity was determined to be 85.0% and positive predictive value was 74.3%. The MPA is based on the number of encounter diagnoses with migraine, prescription orders for migraine, and whether specialty care for migraine was sought. MPA scores greater than 10 indicate a high probability of having migraine. MPA scores were calculated based on 5-years of longitudinal EHR data (MPA_5Y_) to identify PC patients with a history of care for migraine over the past 5 years and, separately, using the most recent 2-years of longitudinal EHR data (MPA_2Y_), to identify patients with recent care for migraine (Fig. 1).

#### Patient survey and survey data

A stratified random sample of eligible PC patients were invited to complete a survey about headache and migraine history, symptoms, treatment and comorbidities among other data. See Additional file 1: Table S1 for survey details. The sampling strata were defined by probability of having migraine, whether care for migraine was recent (i.e., previous 2-years), and by whether, in addition to PC visits, the patients sought care for migraine from a neurologist (Fig. 1). The five sampling strata (Table 1), denoted A through E, were defined as described in Table 1 for PC population who had at least 1 clinical encounter in the 12-months before the survey:

- *Sampling Group A* Recent Neurology Care for Migraine: Had care for migraine in the past 5 years (MPA_5Y_ > 10) and in the past 24-months (MPA_2Y_ > 10) and had care from a neurologist in the previous 5-Year MPA period.
- *Sampling Group B* Recent Non-neurologic care for Migraine: Similar to “A”, but never had care from a neurologist.
- *Sampling Group C* Remote Migraine Care: Had care for migraine in the past 5 years (MPA_5Y_ > 10) but not in the past 24 months (MPA_2Y_ ≤ 10).
- *Sampling Group D* Recent Care for Headache NOS: Had care for headache NOS in the past 5 years, but not for migraine (MPA_5Y_ < 10).
- *Sampling Group E* No Care for Headache: Did not have care for either migraine or any type of headache in the past 5 years.

**Table 1:** Patient strata for the selection of a stratified random sample of patients, Migraine Signature Survey

Strata description for sampling groups	5-year and 2-year MPA scores		EHR headache diagnosis		1 + migraine encounter with a neurologist	Size of source sample	Number sent a survey	Response percent (n)	Sampling weights^1^
	MPA_5Y_ > 10	MPA_2Y_ > 10	Headache NOS	Migraine					
A: Recent migraine care from neurology	Yes	Yes		Yes	Yes	14,139 (1.45%)	2368	17.6% (404)	35.0
B: Recent migraine care from primary care					No	36,722 (3.78%)	2434	14.1% (341)	107.7
C: Past but no recent care for migraine	Yes	No				23,645 (2.43%)	5333	10.8% (575)	41.1
D: Headache care, NOS	No		Yes	No	No	69,704 (7.17%)	7737	5.2% (399)	174.7
E: No care for headache			No	No	No	828,325 (85.17%)	10,396	5.6% (586)	1413.5
Total						972,535 (100%)	28,268	8.2% (2305)	

^1^Sample weight for each strata is calculated as: size of source sample/number of respondents

To be eligible for analysis of the survey data, patients had to have at least 1 encounter of any type and for any condition in the 12-month period before September 13, 2018 when the first email was sent inviting participation in a web-based questionnaire (Additional file 1: Table S1). The questionnaire asked about headache and migraine frequency and symptoms, comorbidities, and patient reported outcomes, including the Migraine Disability Assessment Scale (MIDAS), as detailed below [28, 29].

The survey also included the American Migraine Study/American Migraine Prevalence and Prevention Study (AMS/AMPP) migraine diagnostic screener used to identify survey respondents meeting International Classification for Headache Disorders (ICHD) criteria for migraine [30]. Patients were invited to participate in the survey if they had an email in their EHR. Eligible patients from each stratum who were invited to participate could access a link within the email to the consent form and the questionnaire. The last invitation email was sent on December 8, 2018. Stratums A, B, and C were intentionally over-sampled to ensure that enough patients with migraine participated in the survey. Moreover, stratum specific response rates were monitored and email invitations to new additional patients were sent to ensure that stratum specific quota were met.

### Definition of migraine diagnosis, treatment status, and migraine-related disability

#### EHR data

EHR data were used to define past (MPA_5Y_) and recent (MPA_2Y_) status on use of care for migraine and probability of having migraine and to specifically identify orders for acute and preventive migraine treatments.

#### Clinically diagnosed migraine

EHR documentation of use of care for migraine was used to identify those with clinically diagnosed migraine. An MPA_5Y_ score greater than 10 was used to identify PC patients who had migraine care and an MPA_2Y_ greater than 10 was used to identify patients with more recent migraine care.

#### Migraine treatment order and adjudication status

EHR data were used in the 12-month period before a completed survey was returned to identify migraine indicated acute and preventive treatment orders for each respondent. A randomly assigned 12-month period was used to extract the same data on non-respondents, where the distribution of the 12-month time periods was the same as that for respondents. Longitudinal EHR data for the 12-month period were specifically used to determine if a patient had been prescribed at least one acute treatment for migraine, at least one preventive treatment for migraine, the total count of prescription acute and preventive treatment orders, and the specific class of medications prescribed. We identified prescriptions that were ordered as a result of encounters with a headache diagnosis. Acute treatments were categorized as non-narcotic analgesics, narcotic analgesics, triptans, and other migraine specific treatments, and preventive treatments were categorized as beta-blockers, calcium channel blockers, antidepressants, anticonvulsants, and onabotulinumtoxinA. Most patients who were prescribed an acute or preventive treatment for migraine only received one to three prescriptions, where the overall distribution is highly right skewed. As such, for analysis, prescription order status was defined using a binary variable that distinguished 1–2 orders from 3 or more orders.

#### Migraine related disability

Survey data were used to separately identify respondents with active migraine and to assess disability impact of migraine.

*Active migraine* status was defined from survey responses by applying ICHD-3 criteria for migraine with or without aura to the AMS/AMPP migraine diagnostic questionnaire data [30, 31]. The screener has been previously validated and captures data relevant to the International Classification of Headache Disorders- 3rd edition (ICHD-3) criteria for migraine including headache pain characteristics, exacerbation by routine activity, and associated symptoms [30, 31].

*Migraine related disability* was assessed with the 5-item Migraine Disability Assessment (MIDAS) scale, a 5-item scale assessing missed and reduced productive days at work, school, or home as well as social and leisure activities during the previous 3 months due to headache [28, 29]. Responses were summed and grouped to identify disability by 4 grades: little or none (score of 0–5, Grade I), mild (score of 6–10, Grade II), moderate (score of 11–20, Grade III), and severe (score of ≥ 21, Grade IV) [28, 29]. MIDAS Grade is often used in clinical trials and specialty care practices as a measure of impact of migraine on functioning and as an indication of treatment need where a higher MIDAS Grade indicates a greater need for acute treatments and, in particular, for preventive treatments. Due to the skewed distribution, we dichotomized MIDAS into a low disability group (Scores 0–10, Grades I–II) and moderate-high disability group (Score 11+, Grades III–IV).

### Statistical methods

Analyses were completed to determine the relation between current migraine-related disability status (low vs. moderate-high disability), as documented from survey responses, and EHR-based estimates of the proportion of patients prescribed acute and preventive migraine treatments. The relations were estimated without any corrections, with correction for sampling weights, and then with correction for both sampling weight and non-response bias, using the methods described below. Each individual who completed a survey was assigned a sampling weight that was derived for each strata as the inverse of the sampling fraction for that strata or 1.0 divided by the ratio of the number of respondents in a specific strata divided by the number of individuals from the source population in that respective strata (Table 1). The sampling weight is the size of the source sample divided by the number of respondents within that stratum and it is influenced by both the proportion of individuals in a stratum who were sent surveys and the proportion that completed surveys.

To correct for non-response bias we estimated response propensity scores using standard logistic regression models, where the dependent variable was response status (i.e. response = 1, non-response = 0) to the survey [32–35]. Independent variables were derived from EHR data in the 12-months before the patient response on demographics, migraine comorbidities, and migraine related variables specific to diagnoses, use of care, and medication orders. All analyses were stratified by sampling strata. The response propensity for each individual was estimated from the final model, along with the prediction error. The final weight (i.e. fully adjusted) that was assigned to each respondent was the product of the inverse of the strata-specific sampling fraction and the inverse of the individual predicted response propensity. The non-response bias corrected measures were estimated using weighted outcomes among respondents, and standard errors (SE) were estimated from bootstrapping with 1000 iterations [36].

Analyses were completed in three steps. First, we describe demographics and co-morbidities of the total source population along with that of survey respondents and non-respondents. Second, using EHR data from respondents only, we derived sampling weighted estimates for six demographics and comorbidities. We then corrected for non-response bias. These analyses were completed to validate that the adjusted estimates for all survey participants were similar to proportions in the total source population (Table 2). The proportion difference between respondents and non-respondents was assessed using the Chi-square test, and the proportion difference of estimates in the last two columns in Table 2 (i.e., estimated proportions corrected for sampling weights and sampling weights + response bias) versus the source population were tested using a proportion test for partially overlapped samples [37]. The same analyses were then completed for respondents who had EHR diagnosis of migraine from strata A–C to estimate the proportion who were prescribed an acute or preventive medication (Table 3).

**Table 2:** Demographic features and clinical diagnoses percentages by survey response status and without and with corrections for Sampling Weight and for Sampling Weight and Response Bias ^‡^

Variable	Category	Total source population^1^ % (SE) (N = 972,535)	Survey population from strata A-E		Survey population corrected estimates from strata A-E	
			Respondent^1^% (SE) (n = 2305)	non –respondents^1^ (SE) (n = 25,963)	Corrected for sampling weight % (SE) (n = 2305)	Corrected for sampling weight and response bias % (SE) (n = 2305)
*Percent distribution*						
Sex	Female	61.5% (0.05%)	75.2% (0.9%)^†,^*	67.3% (0.3%)	67.0% (0.2%)^††^	60.0% (0.05%)
Age	18–29	10.0% (0.03%)	17.7% (0.8%)^†,^*	11.2% (0.2%)	19.5% (0.2%)^††^	10.8% (0.03%)
	30–44	27.5% (0.05%)	33.6% (1.0%)^†,^*	28.9% (0.3%)	31.2% (0.2%)^††^	27.1% (0.05%)
	45–64	37.8% (0.05%)	36.4% (1.0%)^†,^*	42.6% (0.4%)	35.1% (0.2%)^††^	38.0% (0.05%)
	≥ 65	24.6% (0.04%)	12.2% (0.7%)^†,^*	17.4% (0.2%)	14.2% (0.1%)^††^	24.1% (0.04%)
Hispanic	No	76.1% (0.04%)	79.6% (0.8%)^†,^*	76.3% (0.3%)	79.1% (0.2%)^††^	76.3% (0.04%)
	Yes	11.2% (0.03%)	9.7% (0.6%)^†,^*	11.9% (0.2%)	8.3% (0.1%)^††^	11.1% (0.03%)
	Other	12.7% (0.03%)	10.8% (0.7%)^†,^*	11.7% (0.2%)	12.6% (0.1%)	12.6% (0.03%)
Race	Asian	16.8% (0.04%)	10.9% (0.6%)^†,^*	15.8% (0.2%)	16.0% (0.1%)	16.5% (0.04%)
	Black	3.2% (0.02%)	1.9% (0.3%)^†,^*	3.7% (0.1%)	1.4% (0.05%)^††^	3.1% (0.02%)
	White	54.6% (0.05%)	66.1% (1.0%)^†,^*	55.3% (0.3%)	62.3% (0.2%)^††^	54.8% (0.05%)
	Other	25.4% (0.04%)	21.1% (0.8%)^†,^*	25.2% (0.3%)	20.3% (0.2%)^††^	25.6% (0.04%)
Marital status	Married/significant other	58.7% (0.05%)	54.1% (1.0%)^†,^*	57.4% (0.3%)	54.1% (0.2%)^††^	58.9% (0.05%)
	Single	21.9% (0.02%)	28.7% (0.9%)^†,^*	25.5% (0.3%)	26.8% (0.2%)^††^	22.1% (0.04%)
	Divorced/separated	4.5% (0.02%)	6.1% (0.5%)^†,^*	5.6% (0.1%)	5.5% (0.09%)^††^	4.6% (0.02%)
	Widowed	3.9% (0.02%)	1.1% (0.2%)^†,^*	2.0% (0.09%)	0.9% (0.04%)^††^	3.8% (0.01%)
	Other/unknown	11.0% (0.03%)	10.0% (0.6%)^†^	9.6% (0.2%)	12.6%^††^ (0.1%)^††^	10.6% (0.03%)^†††^
*Percent of patients with a diagnosis*						
EHR diagnosis^2^	Migraine	7.7% (0.03%)	57.3% (1.0%)^†,^*	33.9% (0.3%)	16.7%^††^(0.02%)	7.7% (0.03%)
	Depression	2.3% (0.02%)	3.8% (0.4%)^†,^*	3.2% (0.1%)	2.4% (0.06%)	2.2% (0.01%)
	Anxiety	5.1% (0.02%)	8.2% (0.6%)^†,^*	7.4% (0.2%)	5.9%^††^ (0.1%)	5.1% (0.02%)
	Autoimmune	1.4% (0.01%)	2.3% (0.3%)*	1.9% (0.08%)	1.5% (0.05%)	1.4% (0.01%)
	Other Pain disorders	3.0% (0.02%)	4.9% (0.5%)^†,^*	4.0% (0.1%)	3.8%^††^ (0.08%)	2.9% (0.02%)
	Respiratory	4.1% (0.02%)	5.8% (0.5%)^†,^*	5.2% (0.1%)	4.9%^††^ (0.09%)	4.0% (0.02%)
	Cardiovascular	4.3% (0.02%)	1.8% (0.3%)^†,^*	3.2% (0.1%)	2.8%^††^ (0.07%)	4.2% (0.02%)
	Neurologic	0.7% (0.01%)	1.2% (0.2)*	1.0% (0.06%)	0.8% (0.04%)	0.7% (0.01%)
	Cerebrovascular	0.02% (0.005%)	0.2% (0.1%)*	0.16% (0.02%)	0.05% (0.01%)	0.01% (0.005%)

^‡^Covariates in the response/non-response model include: age, sex, race/ethnicity, marital status, comorbidities, acute medication orders (0, 1–2 orders, 3+ orders), preventive medication orders (0, 1–2 orders, 3+ orders)

not adjusted for either sampling weight or for response bias

Relevant ICD-9 codes appear 2+ times in a 1-year period as an encounter diagnosis or a medication order indication

^†^Statistically significant comparing respondents versus non-respondents

*Statistically significant comparing respondents versus source population

^††^Statistically significant comparing adjusted for sample weight estimate versus source population

^†††^Statistically significant comparing full adjustment estimate versus source population

**Table 3:** Diagnosed migraine patients with a migraine specific prescription order 12-months before their completed survey ^‡^

Specific treatment	All primary care patients diagnosed with migraine % (SE) (N = 74,506)	Survey population with EHR diagnosed migraine		Survey population with diagnosed migraine corrected estimates	
		Respondents* % (SE) (n = 1320)	Non-respondents* % (SE) (n = 8815)	Corrected for sampling weight** % (SE) (n = 1320)	Corrected for sampling weight and response bias**^,†††^ % (SE) (n = 1320)
*Acute medication orders*					
All acute prescription treatments	**26.4% (0.2%)**	**33.3%**^**†**^** (1.3%)**	**20.4% (0.4%)**	**37.2%**^**††**^** (0.5%)**	**26.2% (0.2%)**
Non-narcotic analgesics					
1–2 orders	1.5% (0.1%)	2.1%^†^ (0.4%)	1.4% (0.1%)	1.6% (0.1%)	1.6% (0.1%)
3 + orders	0.8% (0.09%)	1.0%^†^ (0.3%)	0.7% (0.09%)	1.0% (0.1%)	0.8% (0.03%)
Narcotic analgesics					
1–2 orders	1.8% (0.1%)	3.2%^†^ (0.5%)	1.6% (0.1%)	3.2%^††^ (0.2%)	2.0%^†††^ (0.05%)
3 + orders	1.8% (0.1%)	2.6%^†^ (0.4%)	1.7% (0.1%)	2.6%^††^ (0.2%)	1.8% (0.05%)
Triptans					
1–2 orders	14.2% (0.3%)	20.8%^†^ (1.1%)	13.0% (0.4%)	24.1%^††^ (0.4%)	14.3% (0.1%)
3 + orders	4.1% (0.2%)	6.6%^†^ (0.7%)	3.7% (0.2%)	7.5%^††^ (0.3%)	4.1% (0.08%)
Other migraine-specific prescriptions					
1–2 orders	1.2% (0.1%)	2.6%^†^ (0.4%)	1.0% (0.1%)	2.5%^††^ (0.2%)	1.1% (0.04%)
3 + orders	0.2% (0.04%)	0.4%^†^ (0.2%)	0.2% (0.04%)	0.4%^††^ (0.06%)	0.2% (0.01%)
*Preventive medication orders*					
All preventive treatments	**12.0% (0.1%)**	**17.1%**^**†**^** (1.0%)**	**11.2% (0.3%)**	**16.8%**^**††**^** (0.4%)**	**11.6% (0.1%)**
Beta blockers, any					
1–2 orders	2.3% (0.2%)	3.1%^†^ (0.5%)	2.1% (0.1%)	3.2%^†^ (0.2%)	2.4% (0.06%)
3 + orders	0.5% (0.07%)	0.9%^†^ (0.3%)	0.5% (0.08%)	0.9%^††^ (0.09%)	0.5% (0.02%)
Calcium channel blockers, any					
1–2 orders	0.9% (0.09%)	1.6%^†^ (0.3%)	0.8% (0.09%)	1.4%^††^ (0.1%)	0.9% (0.03%)
3 + orders	0.2% (0.04%)	0.3% (0.1%)	0.2% (0.04%)	0.3% (0.06%)	0.2% (0.02%)
Antidepressants, any					
1–2 orders	3.1% (0.2%)	4.5%^†^ (0.6%)	3.0% (0.2%)	4.9%^††^ (0.2%)	3.1% (0.06%)
3 + orders	1.0% (0.1%)	1.6%^†^ (0.3%)	0.9% (0.1%)	1.5%^††^ (0.1%)	0.9% (0.03%)
Anticonvulsants, any					
1–2 orders	3.9% (0.2%)	5.7%^†^ (0.6%)	3.6% (0.2%)	5.1%^††^ (0.2%)	3.8% (0.07%)
3 + orders	1.6% (0.1%)	2.3%^†^ (0.4%)	1.5% (0.1%)	2.1%^††^ (0.1%)	1.5% (0.04%)
OnabotulinumtoxinA, any					
1–2 orders	1.0% (0.1%)	1.8%^†^ (0.4%)	0.9% (0.1%)	1.6%^††^ (0.1%)	0.9% (0.03%)
3 + orders	0.06% (0.02%)	0.08% (0.08%)	0.06% (0.03%)	0.06% (0.02%)	0.05% (0.01%)

The bold is used to distinguish the total for acute and preventive treatment proportions from the proportions for specific classes of acute and preventive medications

^‡^Covariates in the response/non-response model include: age, sex, race/ethnicity, marital status, comorbidities, acute medication orders (0, 1–2 orders, 3 + orders), preventive medication orders (0, 1–2 orders, 3 + orders)

*Not adjusted for strata distribution

**In addition, adjusted for strata distribution

^†^Statistically significant comparing respondents versus population

^††^Statistically significant comparing adjusted for sample weight estimate versus source population

^†††^None of variables is statistically significant comparing full adjustment estimate versus source population

Respondents from strata D and E were excluded from the latter analysis because the focus was on patients with EHR documentation of care for migraine. Treatment status was estimated as the proportion of respondents prescribed acute and preventive treatments in the 12-month period before the web-survey was completed. A final analysis was then completed using respondent data from strata A–D who met migraine criteria to determine the relation of MIDAS Grade and migraine treatment status. Estimates were stratified by those who had MIDAS Grades of I–II versus III–IV (Table 4), where a binomial test was applied for each row in Table 4 for the corrected proportions compared to the uncorrected proportions in the last two columns. We hypothesized that the corrected proportion would not differ from the uncorrected proportions (as the expected value) in Table 4. The binomial tests in these comparisons may over-estimate the significance of differences given that the assumption of independence after correction for the inverse propensity might not hold and where the variance of corrected estimate is likely to be underestimated. In addition, we accounted for multiple comparison for each acute and preventive medication using the Bonferroni correction (i.e. alpha = 0.05/4) (last two columns in Table 4).

**Table 4:** Survey diagnosed migraine patients by MIDAS grade with prescription orders 12-months before their completed survey

Prescription medication orders	No corrections		Corrected for sampling weight only from Strata A–D*		Corrected for sampling weight and response bias from Strata A–D*	
	MIDAS I–II N = 729% (SE)	MIDAS III–IV N = 791% (SE)	MIDAS I–II N = 729% (SE)	MIDAS III–IV N = 791% (SE)	MIDAS I–II N = 729% (SE)	MIDAS III–IV N = 791% (SE)
*Acute treatment orders 12 months before the completed survey*						
All acute treatment prescriptions	**28.6% (2.0%)**	**37.7% (1.8%)**	**32.3% (0.2%)**	**37.3% (0.2%)**	**24.8%**^†^ **(0.06%)**	**26.6%**^††^ **(0.06%)**
Non-narcotic analgesics**						
1–2 orders	1.4% (0.5%)	2.5% (0.6%)	1.2% (0.4%)	1.5% (0.04%)	2.5%^†^ (0.02%)	1.0%^††, †††^ (0.01%)
3 + orders	0.4% (0.3%)	1.3% (0.4%)	0.3% (0.02%)	1.4% (0.04%)	0.3% (0.01%)	1.1%^†††^ (0.01%)
Narcotic analgesics**						
1–2 orders	2.8% (0.7%)	3.8% (0.7%)	2.5% (0.06%)	3.7% (0.07%)	1.5%^†^ (0.02%)	2.7%^††,†††^ (0.02%)
3 + orders	1.4% (0.5%)	3.6% (0.7%)	1.5% (0.04%)	2.8% (0.06%)	1.1% (0.01%)	2.2%^††,†††^ (0.02%)
Triptans**						
1–2 orders	20.3% (1.8%)	21.5% (1.6%)	23.4% (0.2%)	23.5% (0.2%)	17.0%^†^ (0.05%)	18.1%^††,†††^ (0.05%)
3 + orders	4.3% (0.9%)	8.7% (1.1%)	5.3% (0.08%)	8.3% (0.1%)	3.1%^†^ (0.02%)	6.1%^††,†††^ (0.03%)
Other migraine-specific treatments**						
1–2 orders	0.6% (0.3%)	4.1% (0.7%)	0.6% (0.03%)	3.1% (0.06%)	0.2%^†^ (0.01%)	1.4%^††,†††^ (0.02%)
3 + orders	0.2% (0.2%)	0.6% (0.3%)	0.2% (0.02%)	0.5% (0.03%)	0.06% (0.005%)	0.2%^††,†††^ (0.01%)
*Preventive treatment orders 12-months before the completed survey*						
All preventive treatments prescriptions	**11.8% (1.4%)**	**21.6% (1.6%)**	**12.0% (0.1%)**	**17.7% (0.1%)**	**8.1%**^†^ **(0.04%)**	**12.0%**^††^ **(0.04%)**
Beta blockers, any						
1–2 orders	2.8% (0.7%)	3.8% (0.7%)	2.7% (0.06%)	3.7% (0.07%)	2.4% (0.02%)	2.7%^††^ (0.02%)
3 + orders	0.2% (0.2%)	1.6% (0.5%)	0.3% (0.02%)	1.0% (0.04%)	0.2% (0.01%)	0.5%^††^ (0.01%)
Calcium channel blockers, any						
1–2 orders	1.6% (0.5%)	1.5% (0.5%)	1.3% (0.04%)	1.0% (0.04%)	0.6%^†^ (0.01%)	0.6%^††^ (0.01%)
3 + orders	0.2% (0.2%)	0.3% (0.2%)	0.1% (0.01%)	0.4% (0.02%)	0.03% (0.002%)	0.2% (0.01%)
Antidepressants, any						
1–2 orders	3.6% (0.8%)	5.1% (0.8%)	4.1% (0.07%)	4.7% (0.08%)	2.3%^†^ (0.02%)	2.6%^††^ (0.02%)
3 + orders	1.0% (0.4%)	2.2% (0.6%)	1.0% (0.04%)	1.7% (0.05%)	0.5%^†^ (0.01%)	1.0%^††,†††^ (0.01%)
Anticonvulsants, any						
1–2 orders	3.7% (0.8%)	7.4% (1.0%)	3.5% (0.07%)	5.4% (0.08%)	2.7%^†^ (0.02%)	3.9%^††,†††^ (0.03%)
3 + orders	1.4% (0.5%)	3.2% (0.7%)	1.3% (0.04%)	2.1% (0.05%)	0.6%^†^ (0.01%)	1.9%^††,†††^ (0.02%)
Onabotulinumtoxin A, any						
1–2 orders	0.8% (0.4%)	2.6% (0.6%)	0.7% (0.03%)	1.8% (0.05%)	0.3%^†^ (0.01%)	1.0%^††^ (0.03%)
3 + orders	0.2% (0.2%)	0%	0.2% (0.02%)	0%	0.1% (0.002%)	0%

The bold is used to distinguish the total for acute and preventive treatment proportions from the proportions for specific classes of acute and preventive medications

*Also adjusted for strata distribution

^†^Statistically significant comparing fully adjusted versus no correction for MIDAS I–II

^††^Statistically significant comparing fully adjusted versus no correction for MIDAS III–V

^†††^Statistically significant comparing fully adjusted MIDAS I–II versus MIDAS III–V

**Multiple comparison: statistically significant comparing fully adjusted MIDAS I–II versus MIDAS III–V

Analyses were performed in SAS (v9.4, SAS institute, Inc, Cary, NC). All statistical tests were two-sided with a p-value of less than 0.05 considered as a cut-off for statistical significance. We used the Proc Surveyfreq SAS procedure to account for weight.

## Results

Figure 1 describes the source population of eligible primary care patients and the number and percent of patients assigned to the five strata or sampling groups,  summarized in Fig. 1 and Table 1. A total 972,535 patients met eligibility criteria, having at least one episode of care in the 12 months before September 13, 2018 (Table 1) for any reason; 28,268 PC patients were randomly selected from the five strata and invited to participate in the web-survey, where 2305 (8.9%) responded. Response rates varied from 17.6% for stratum A to 5.2% for stratum D (Table 1). Results are first summarized for the total population of adult PC patient on demographics and diagnoses (Table 2) and then for patients with diagnosed migraine on the proportion with orders for acute and preventive treatments (Table 3). Tables 2 and 3 summarize estimates for the total relevant source population with estimates corrected for sampling weight and non-response bias. Table 4 describes the relation between MIDAS score derived from the survey and migraine prescription order status in the previous year without and with corrections for sampling weight and non-response bias.

### Demographic and selected diagnostic features

The stratified random sample of survey respondents differed from the total source population on all demographic variables and all diagnostic variables summarized in Table 2. Separately, compared to non-respondents, respondents differed significantly on almost all of the demographic and diagnostic variables.

#### Corrected estimates for demographic and EHR diagnosis

Correction for sampling weight (Table 2) reduced differences in estimates for respondent EHR variables compared to the source population for all demographic and EHR diagnostic variables, but most of these variables were still significantly different from the source population except depression, autoimmune, neurologic and cerebrovascular conditions. After correcting for both sampling weight and non-response bias (Table 2, the rightmost column), estimates of distributions by demographic factors and clinical diagnoses were very similar and none of the comparisons to the total source population were significantly different except for one level in marital status (i.e. other/unknown).

#### Corrected estimates for acute and preventive treatments orders

We estimated the proportion of patients who were prescribed an acute treatment for migraine and, separately, the proportion prescribed a preventive treatment for migraine (Table 3). Though parallel to the analysis in Table 2, Table 3 includes data on patients from strata A, B, and C specific to migraine prescription orders. Patients from strata D and E were excluded because they did not have EHR documentation of migraine or a prescription treatment order for migraine. Compared to respondents, non-respondents were considerably less likely to have a prescription order in their EHR in the year before the survey for either an acute (33.3% vs. 20.4%, *p* value < 0.001) or preventive medication (i.e., 17.1% vs. 11.2%, *p* value < 0.001).

When corrected for sampling weights, the estimated proportion of survey respondents with an acute prescription treatment order was substantially greater than the source population estimate (37.2% vs. 26.2%) and greater than the uncorrected estimate (33.3%). The sampling weight corrected proportion with a preventive treatment order was also significantly greater than the source population proportion estimate (16.8% vs. 12.0%).

When we added a correction for non-response bias (Table 3, rightmost column), estimates compared to the source population dramatically improved (Table 3, leftmost column) for acute (26.2% vs. 26.4%) and preventive treatments (11.6% vs. 12.0%). None of the non-response bias corrected estimates for the overall acute and preventive medication orders or for medication specific orders were significantly different from those of the source population.

#### Corrected estimates for acute and preventive treatment orders by MIDAS grade

Survey data from respondents in strata A-D were used to understand the relation of MIDAS Grade and prescription medication orders. Among the 1719 survey respondents that met ICHD criteria for migraine, 1520 (88%) completed the MIDAS questionnaire (Additional file 1: Table S2). Completion rates for the MIDAS questionnaires varied by strata from 92.7% for stratum B to 81.0% for stratum D (Additional file 1: Table S2).

Patients with MIDAS Grades III–IV had more prescription orders for both acute and preventive treatments overall and for specific treatment classes than those with MIDAS Grades I–II (Table 4). Among those in MIDAS Grade I–II, the adjustment for sampling weights increased the estimated proportion that were prescribed an acute medication but had little or no effect on the estimated proportion with a preventive treatment order. Among those with MIDAS Grades III–IV, the sampling fraction corrected estimate was unchanged for the proportion prescribed an acute treatment and was lower and improved for the proportion prescribed a preventive treatment (Table 4).

When correcting for both sampling weight and response bias the estimated proportions with an acute or a preventive treatment were significantly lower than the uncorrected estimates for both the MIDAS Grades I–II and III–IV groups (Table 4, last two columns). The differences were larger, however, for patients in the MIDAS Grade III–IV group where the corrected estimates were substantially lower than the uncorrected estimates. MIDAS Grade III–IV patients were significantly more likely than MIDAS Grade I–II patients to have orders for acute treatment, for anticonvulsants (i.e., both 1–2 and 3+ orders), and for 3+ orders of anti-depressant preventive treatments. Multiple comparison adjusted testing revealed that each acute treatment was more likely to be prescribed to MIDAS Grade III–IV patients than to MIDAS Grade I–II patients, but no differences were observed by MIDAS Grade for preventive medication orders.

## Discussion

Assessing the value of care for many diseases can be challenging if patient reported information on disease onset, progression, severity, and other factors is essential to evaluating quality of care. This is especially true for conditions like migraine and the diversity of other chronic diseases with episodic manifestations for which there are no objective clinical or laboratory measures of disease status, severity or control [38]. PROs and, more generally, self-reported experience is central to evaluating population level care gaps. We consider the limitations of using administrative claims or EHR data (e.g., medication prescriptions) versus self-reported data (e.g., MIDAS) to understand quality of care for migraine and the unique advantages that come from combining these two data sources.

Population-based surveys are often used to understand the epidemiology of disease and related use of care. These types of surveys usually include clinically validated questionnaires to standardize detection of active disease and the measurement of disease severity, whether or not an individual sought care for the specific disease, and whether or not it was diagnosed. These approaches have been particularly useful for migraine as a substantial minority of people with migraine do not seek medical care and may not receive a medical diagnosis [6, 39]. Quality of care gaps can also be quantified with self-reported information on the experience of care. But survey data of health conditions are often inherently limited because only a minority of those invited will participate. Moreover, the likelihood of participation is usually related to having the disease of interest, to the severity of disease, and to the use of care [15–17]. Self-report is also prone to selective recall and other types of biases that may yield a distorted understanding of the relation between disease severity and utilization of care. The results of this study reveal response biases (Table 2, respondents vs non-respondents) that are consistent with previous surveys where females, non-Hispanics, Whites, and those with a greater disease burden are more likely to participate [15–18].

EHR or medical claims and pharmacy data reveal utilization of those who seek care for a specific disease. But ascertainment is often incomplete because many diseases are difficult to detect using diagnostic codes. Migraine is often assigned a non-specific diagnostic code (e.g., Headache NOS) [40]. In our study, the source population headache NOS group accounted for 48% (i.e., 69,704/144,201, Table 1) of primary care patients with a primary headache diagnosis [41]. The survey data in this report indicated that a substantial proportion of those with headache NOS have moderate to severe migraine, confirming prior work [41]. In addition, for migraine, in particular, survey data indicate that a substantial minority report never having sought care for migraine and, accordingly, would never be identified from EHR data [4, 6]. Finally, EHR data lack information on disease onset, severity, progression, and other meaningful outcomes for conditions like migraine deemed essential to identifying care gaps [42].

The complementary strengths and weaknesses of survey and EHR data offer a synergistic and powerful means of gaining a comprehensive and accurate assessment of disease burden and patterns of care within a health system. The synergy comes from the way in which EHR data, available on all patients, can be used to eliminate problems with recall bias and, importantly, overcome selection bias challenges from survey non-response. We specifically focused on migraine for this study because it is representative of many other symptomatic and burdensome diseases common to adolescents and working age populations where people often do not seek care, do not receive a diagnosis when they do seek care, or are under-treated [3–8].

We validated the method of adjustment for non-response bias using known source population data that included demographics, diagnosed comorbid diseases, and prescriptions for migraine or headache orders. Even with the relatively low response rates in each strata the corrected estimates using data from survey respondents were similar to the source population estimates (Tables 2, 3). Survey respondents were more likely to respond if they had at least one acute or preventive treatment order overall and for each of the medication specific classes. The response bias differences are in the expected direction given what is known about selective participation of those with more severe disease and when the purpose of the survey is known in advance.

Direct comparisons to previous studies on the use of prescription medications are difficult. Studies differ substantially in the source of data and in the factors that directly influence the estimated proportion of patients using a prescription medication [24, 25, 43, 44]. Studies also differ in the selection criteria used for study participation. Some focus on newly diagnosed patients while others enrolled patients from particular care settings such as the emergency department. Look back periods for assessing use of prescription medications also widely varied as did the means of assessing treatments used (e.g., pharmacy claims data or survey self-report).

The American Prevalence and Prevention Study (AMPP) offers the most relevant comparative survey data on prescription medications used by those with migraine [5]. AMPP had a higher response rate than more recent web-surveys conducted in the US. The AMPP Study is a longitudinal national survey that used the same validated diagnostic screener as was used in our study to identify adults meeting ICHD diagnostic criteria for migraine. Among ***all*** AMPP survey respondents that met active ICHD migraine criteria, 20.1% reported current use of an acute prescription treatment and 13% reported current use of a preventive medication for migraine. The percentage for current use of preventive treatment excludes coincidental use for other health problems as we did in our study. But our analysis of prescription medication for migraine was confined to primary care patients with a physician diagnosis of migraine, whereas the AMPP study included participants whether or not they reported a medical diagnosis and whether or not they had a recent episode of medical care for migraine [42]. Comparable numbers for the AMPP Study sample can be derived by limiting the denominator to the 56.2% of those with ICHD criteria for migraine that self-reported having received a medical diagnosis of migraine. Of these, 35.9% were using an acute prescription treatment and 23.0% were using a preventive treatment. By comparison, among those with a medical diagnosis of migraine in our study (Table 3), the corrected comparable estimates are considerably lower for acute prescription medications (i.e., 26.2% vs. 35.9% in AMPP) and preventive medications (11.6% vs. 23% in AMPP). The substantially lower estimate using Sutter Health MSS data may indicate that the actual use of prescription treatments by people with migraine is overestimated in population surveys. This could be explained by selective participation of those with more frequent, severe and disabling migraine who have been prescribed a treatment.

Medication orders for acute and preventive migraine treatments were greater for those with MIDAS Grade III–IV than for those with MIDAS Grade I–II, particularly for preventive treatment (Table 4). This trend was expected and is consistent with a previous study [44]. The differences between the sample-weighting-corrected estimates and the fully corrected estimates are striking. The fully corrected estimates for MIDAS Grade III–IV, in particular, are substantially lower than either the uncorrected or the sample-weighting-corrected estimates. Moreover, after accounting for multiple comparisons, no differences were observed by MIDAS Grade for preventive medication orders. This finding suggests that that individuals with MIDAS Grade III and IV migraine are more likely to respond to surveys than those in Grades I and II migraines. Though the statistical test (i.e. binomial test in Tables 3, 4) may over-estimate the statistical significance between difference of fully adjusted and unadjusted proportion, the quantity of the proportion difference for each treatment suggest that previous population surveys may substantially overestimate the use of acute and preventive treatments, especially for patients with MIDAS Grade III and IV [44].

## Conclusion

Combining survey and EHR data has many potential applications to evaluating quality of care even when survey response rates are low. Because EHR data are available on all individuals whether or not they respond to a survey, statistical methods serve to adjust for non-response bias in ways that cannot be resolved by traditional approaches to motivating participation (e.g., gift card or other incentives) [15, 16]. While combining survey and EHR data opens many possibilities to gain a richer population level understanding of the quality of care that patients receive, this same approach may offer a more accurate means of gaining a general understanding the epidemiology of a diversity of health problems. Additional research is required to develop methods for routine internal and external validation and to better understand conditions under which substantial non-response bias may persist even after adjusting for propensity to respond.

Finally, we note that patient surveys and EHR analyses are often used as alternative methods to study disease burden and health care delivery. By collecting surveys in patient samples derived from an integrated delivery system, these two approaches can complement each other in many ways. While technically feasible and promising, linking survey data to EHR data raises issues of patient privacy, informed consent, and the development of strategies for optimizing survey participation and representativeness. This paper illustrates the promise of the method and one approach to addressing selection bias.

## Supplementary Information

**Additional file 1.** **Table S1.** Survey data collected from patients in Strata A-D and Stratum E*. **Table S2.** MIDAS Grade distribution by sampling strata.

## Data Availability

Computing codes can be requested and shared upon Sutter IRB and Information security office approval. Due to HIPAA, patient EHR data cannot be shared with researchers outside of Sutter.

## Abbreviations

EHR: Electronic health records
MIDAS: Migraine Disability Assessment Scale
SES: Socio-economic status
PC: Primary care
EPIC: Epic Care
ICD: International classification of diseases
MPA: Migraine probability algorithm
MPA_5Y_: MPA based on five years of longitudinal EHR data
MPA_2Y_: MPA based on most recent two years of longitudinal EHR data
NOS: Not otherwise specified
AMS: American migraine study
AMPP: American migraine prevalence and prevention
ICHD: International classification of headache disorders
HA: Headache
ASC-12: Allodynia symptom checklist
OTC: Over the counter
MTOQ-6: Migraine treatment optimization questionnaire
GAD-7: Generalized anxiety disorder questionnaire
PHQ-8: Patient health questionnaire
PTSD: Post-traumatic stress disorder
MSS: Migraine signature study
