# Supplementary material for: Combining patient reported outcomes and EHR data to understand population level treatment needs: correcting for selection bias in the migraine signature study
Source: J Patient Rep Outcomes. 2021 Dec 18;5:132. doi: 10.1186/s41687-021-00401-2 (PMC8684566; doi:10.1186/s41687-021-00401-2)
Supplement: Supplementary file 1 — Additional file 1. Table S1. Survey data collected from patients in Strata A-D and Stratum E*. Table S2. MIDAS Grade distribution by sampling strata. [file 41687_2021_401_MOESM1_ESM.docx]

**Additional file 1**

| Supplemental Table 1. Survey data collected from patients in Strata A-D and Stratum E* | | | |
| --- | --- | --- | --- |
| **Domain** | **Variables** | A-D | E |
| Demographics | Age, sex, employment status, education completed, marital status, number of people living in household, annual household income, | x | x |
| Smoking | Smoking (lifetime, current) | x | x |
| HA past year | Headache in the past year | x | x |
| Headache days | Past 90 days, past year, past month, ≥1 moment headache freedom in past month | x |  |
| Number of types of headache | Number of types of headache | x |  |
| Number of headache days for each type | Number of headache days in past 90 days for 1. Most severe, 2. Second most severe, 3. Third most severe type of headache | x |  |
|  | ≥5 lifetime attacks of most severe headache | x |  |
| Migraine/Severe Headache Criteria | Current migraine criteria, migraine symptom severity (MSS),  Headache pain intensity, untreated headache duration | x |  |
| Migraine/Headache | age of onset, self-report of physician headache diagnoses | x |  |
| Migraine/Headache | Headache related disability (MIDAS) | x |  |
| Aura | Aura (visual and sensory) | x |  |
| Female hormonal questions | 1. current status: pregnant, breastfeeding, peri or post-menopausal  2. Time since last menstrual period | x |  |
| Allodynia | Cutaneous allodynia (ASC-12) | x |  |
| Type of treatments ever used for most severe type of headache | Acute OTC, Acute prescription, Preventive prescription pharmacologic, Acute and Preventive Injections, Behavioral, Nutraceutical, Neuromodulation | x |  |
| Preventive medication use | Among 7 most commonly prescribed medications at Sutter Health for migraine prevention: Reasons for discontinuation (if applicable); Satisfaction ; and Side effects | x |  |
| Acute medication use | Reasons for discontinuation (if applicable); Satisfaction ; and Side effects | x |  |
| Medication Overuse | Days per month | x |  |
| Treatment Optimization | Treatment Optimization Questionnaire (MTOQ-6) | x |  |
| Anxiety | GAD-7 (Anxiety) | x | x |
| Depression | PHQ-8 (Depression Without Suicidality Item, Question #9) | x | x |
| Panic Disorder | PHQ-Panic (Panic Disorder, Patient Health Questionnaire, panic section) | x | x |
| PTSD | PC-PTSD (Primary Care PTSD Screen, Civilian Version) | x | x |
| Remitted Migraine/ Headache Module | Age of onset of severe headache  Age severe headache at its worst, frequency at that time  Migraine symptom criteria (AMS/AMPP Diagnostic Module)  Self-report of physician diagnosis of multiple headache types |  |  |
| * Stratum E: no headache diagnosis | | | |

| Supplemental Table 2: MIDAS Grade distribution by sampling strata | | | | | | | |
| --- | --- | --- | --- | --- | --- | --- | --- |
| Strata Name  (Sample Size) | Size of Source Sample | Number of Survey Responses | Completed MIDAS  N (%) | MIDAS Grade  N (%^**^) | | Response Weight | |
|  |  |  |  | I-II | III-IV | I-II* | III-IV* |
| A: Recent migraine care from neurology | 14,139 | 404 | 373  (92.3%) | 112  (30.0%) | 261  (70.0%) | 126.2 | 54.2 |
| B: Recent migraine care from primary care | 36,722 | 341 | 316  (92.7%) | 154  (48.7%) | 162  (51.3%) | 238.4 | 226.7 |
| C: Past but no recent care for migraine | 23,645 | 575 | 508  (88.3%) | 241  (47.4%) | 267  (52.6%) | 98.1 | 88.5 |
| D: Headache care, NOS | 69,704 | 399 | 323  (81.0%) | 222  (68.7%) | 101  (31.3%) | 314.0 | 690.1 |
| *Calculated for each stratum as the source sample size/number of response in each MIDAS group  **- The percentage did not include who did not complete MIDAS questionnaire in that stratum | | | | | | | |
